# The Integration of 3D Virtual Reality and 3D Printing Technology as Innovative Approaches to Preoperative Planning in Neuro-Oncology

**DOI:** 10.3390/jpm14020187

**Published:** 2024-02-07

**Authors:** Pablo González-López, Artem Kuptsov, Cristina Gómez-Revuelta, Jaime Fernández-Villa, Javier Abarca-Olivas, Roy T. Daniel, Torstein R. Meling, Juan Nieto-Navarro

**Affiliations:** 1 Department of Neurosurgery, Hospital General Universitario, 03010 Alicante, Spain; drartemkup@gmail.com (A.K.); crgomezrevuelta@gmail.com (C.G.-R.); jaimefernandez3a@gmail.com (J.F.-V.); jabarcaolivas@gmail.com (J.A.-O.); nieto_juanav@gva.es (J.N.-N.); 2 Centre Hospitalier Universitaire Vaudois, 1005 Lausanne, Switzerland; roy.daniel@chuv.ch; 3 Department of Neurosurgery, Rigshospitalet, 92100 Copenhagen, Denmark

**Keywords:** surgical planning, neuroanatomy, virtual reality, 3D printing technology, surgical simulation

## Abstract

Our study explores the integration of three-dimensional (3D) virtual reality (VR) and 3D printing in neurosurgical preoperative planning. Traditionally, surgeons relied on two-dimensional (2D) imaging for complex neuroanatomy analyses, requiring significant mental visualization. Fortunately, nowadays advanced technology enables the creation of detailed 3D models from patient scans, utilizing different software. Afterwards, these models can be experienced through VR systems, offering comprehensive preoperative rehearsal opportunities. Additionally, 3D models can be 3D printed for hands-on training, therefore enhancing surgical preparedness. This technological integration transforms the paradigm of neurosurgical planning, ensuring safer procedures.

## 1. Introduction

Surgical planning appears to be the fundamental pillar when preparing for any neuro-oncology intervention [1]. It permits surgeons not only to analyze and study a patient’s particular anatomy, important neurovascular structures, and the lesion’s architecture and its relationships but also offers a possibility to anticipate multiple intraoperative scenarios. All this knowledge helps the surgeon to be prepared before entering the operating room. To perform neurosurgical interventions as safely as possible, apart from having solid neuroanatomical knowledge, it is crucial to study each patient’s structural nuances as they may differ from the standards. In current practice, this is mainly achieved by carrying out a profound analysis of the preoperative imaging studies [2]. However, the results of the tests are usually presented in two-dimensional (2D) slices, with the surgeons’ task being to study those images and create a mental image in which this 2D information is transformed into the three-dimensional (3D) scenario which will be found once the surgical field is exposed. This is not an easy task, as it requires a large amount of experience, both in analyzing regular 2D images and integrating the “mentally visualized” anatomy with the real one seen during the surgical procedure [3].

Nowadays, constant technological advances offer valuable tools to visualize 3D anatomical structures derived from 2D slices from a patients’ imaging preoperative tests [4]. But this is not only limited to creating 3D reconstructions of a patient’s anatomy but also integrating them into virtual reality (VR) environments [5,6]. Through the implementation of these 3D objects in a virtual and/or augmented reality (AR) visualization system, surgeons can study the relationships between different structures in a more efficient way, from a surgical point of view. This immersive approach allows the surgeon to place a patient’s 3D reconstructions in the different positions in which surgery can be performed, even simulating different surgical approaches to evaluate the assets and disadvantages of each one [7].

The 3D modeling of neuroanatomical structures is not only applicable with VR/AR but can also be printed with the aid of 3D printing technology [8]. Therefore, by printing 3D models, surgical interventions can be practiced [9]. With this, surgeons and trainees can gain manual skills, start their learning curve, face potential intraoperative gaps, and practice different approaches before carrying out a procedure on the patients themselves [10,11].

The preoperative surgical planning paradigm is changing due to the emergence of technology [12,13,14,15,16]. VR systems and 3D printing devices represent powerful tools to push surgical planning in neurosurgery to the highest standards. Therefore, the main objective of this original research is to provide an original and self-created protocol for generating 3D objects from the medical images of brain tumor patients. These objects can be implemented in a VR/AR environment and can be 3D printed, aiming to enhance the safety and efficiency of neurosurgical procedures. Moreover, our results are summarized by illustrating five selected cases.

## 2. Materials and Methods

### 2.1. Image Data Acquisition

**Figure 1:**
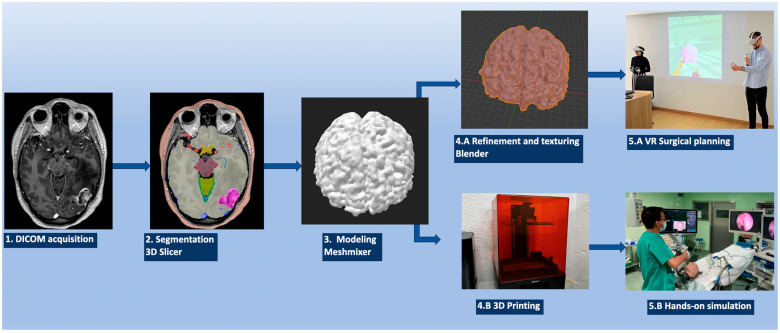
Summary of the original 3D rendering protocol workflow. DICOM files are extracted from the local PACS and imported into the 3D slicer where the segmentation of the different layers is carried out. Each of the .stl files is exported to the Meshmixer software for the modeling process. The 3D segments are then refined and textured through different tools and processes into the Blender version 3.4.1 (3.4.1 2022-12-20) to finally import the .fbx files into the VR system and/or send them to the 3D printer.

The process of creating 3D models, whether for VR visualization or 3D printing, starts with the acquisition of a patient’s radiological data from the imaging tests. For 3D modeling purposes, many different types of medical imaging techniques can be used, depending on the desired anatomical structure to be reconstructed. Among the most utilized image examinations, we recommend computerized tomography (CT) and CT angiography (CTA), magnetic resonance imaging (MRI) including T1 with and without contrast, Time-Of-Flight (TOF), T2, and fluid-attenuated inversion recovery (FLAIR). One important detail to be considered is that all the imaging tests should have 1 to 1.25 mm slices, ensuring the precise creation of the reconstructions, as shown in Figure 1.

Once a patient has undergone the desired imaging test, their Digital Imaging and Communication in Medicine (DICOM) data files are downloaded for further working processes. DICOM is a standardized format of medical images and related documents used in the healthcare sphere for their management, storage, and transmission. The idea behind DICOM is to meet the diverse requirements of the healthcare industry by standardizing the way medical images and related data are managed. Apart from purely graphic information, DICOM associates “metadata”, a valuable compound of features which regular image formats lack. One of the most important ones is the spatial localization and referencing of each single voxel for each single image in the 3D space. This is the key feature that makes 3D reconstructions feasible when working with this format [17].

### 2.2. Object Segmentation

The obtained DICOM data are loaded into the 3D Slicer 5.3.0-2023-07-15 (The Slicer Community, http://www.slicer.org accessed on 15 July 2023). 3D Slicer is a free and open-source software that is meant to visualize, process, segment, and analyze 3D medical and biomedical images [18]. It has plenty of different applications in planning and guiding image-based procedures. For this project, 3D Slicer is mainly used in the “segment editor mode”, a module that helps to segment the desired structure from the imaging test and then export it into a 3D file [19].

In this step, we propose working independently with each “object” to be segmented (tumor, brain, cranial nerves, arteries, veins, bone, skin, cisterns, etc.). Each of the chosen objects is to be manipulated on the image modalities in which the object presents a higher contrast with respect to the surrounding structures so that the processing can be simpler and more accurate. For example, the ideal image for reconstructing the arterial tree is the TOF sequence of an MRI, while the most suitable image for segmenting the bone is a non-contrast CT scan (Figure 1).

The segment editor options range from a fully slice-by-slice manual delineation of the structure to semiautomatic segmentation achieved through tools like “Threshold”, “Grow from seeds” or “Fill between slices”. Also, once segmentation is completed, refinement options for a better 3D visualization of the object are also available, like the “Smoothing” and “Islands” instruments. Once the desired structure is created, the 3D model is exported to a standard triangulated language (STL) file, also known as stereolithography. This format is used to represent 3D surface geometry, using a series of polygons and triangles, mainly in the field of computer-aided design (CAD) and 3D printing.

### 2.3. Sculpting the 3D Objects

The process of creating the 3D object then continues with Autodesk MeshMixer version 3.5.474 (Autodesk Inc., San Rafael, CA, USA). Autodesk MeshMixer is a free user-friendly software (https://meshmixer.com/), mainly developed to clean up and model triangle-based 3D objects. It has multiple features that allow for combining, sculpting, repairing, and refining mesh models [20,21]. Each previously created radio-anatomical object (Obj file) is loaded into this software where the refinement process is carried out mainly through the “Inspector” and “Sculpt” tools. Once the segmentation has been modeled and refined, the different objects can be modeled as desired, simulating the transformation that each might undergo during the surgical procedure depending on the approach, position, and retraction of the structures. Therefore, a craniotomy in the bone structure or the adaptation of the brain or cerebellum after cisternal opening and relaxation can be simulated to make it akin to the real situation that could be expected to happen during the different steps of a surgery. Each object is saved separately, as can be seen in Figure 1.

### 2.4. VR/AR Implementation

Up to this point, each of the segmented anatomical structures is composed of an innumerable number of polygons and triangles and is shown in grayscale, keeping its true position in space. In order to create more realistic objects to be implemented into a VR system, a new process is required. The STL files are then loaded into Blender^®^ for individual 3D modeling work. Blender (Blender Foundation, Amsterdam, The Netherlands, www.blender.org) is an open-source free software that permits further processing of a 3D object. Therefore, the anatomical 3D object’s color, texture, and appearance become closer to the real one. Once the desired 3D model’s form, color, and texture are achieved, the model is exported to a filmbox (FBX) file format. The FBX format is a proprietary file format developed by Autodesk and supports various types of data, including 3D geometry, materials, textures, and lights, among others [22].

This process of creating a 3D object from the initial patient DICOM imaging files is repeated to obtain all the desired anatomical structures as FBX files. Next, all files must be loaded into Oculus Quest Meta glasses. The Oculus Quest is a standalone VR headset that provides an immersive experience without the need to connect it to external sensors or devices [23]. It has built-in tracking and computing engineering making it possible to have freedom of movement in a designated area. The software used to visualize the created 3D objects is Gravity Sketch^®^ (London, UK, www.gravitysketch.com), an individual free software which is mainly used to visualize, model, and design 3D models in a VR environment [24]. Once all the FBX files are loaded into Gravity Sketch, a complete model can be assembled in a 3D virtual space. Note that if all objects are crafted from the same imaging study, they are automatically placed in the correct position, keeping the real anatomical relationships among all the segments. However, structures obtained from different imaging studies, due to differences in their acquisition parameters, are placed in non-anatomical positions, unrelated to one another. This can be corrected by means of anatomical realignment with the Blender software.

Once the above steps have been completed, the virtual 3D visualization of the patient’s anatomy and pathology can be revised and studied. The virtual anatomical specimen can be positioned into a surgical position but also reviewed from different perspectives. Furthermore, Gravity Sketch allows for the creating of a virtual room for more than one user, enabling two or more surgeons and trainees from different locations to join, study, and discuss the surgical plan to carry out a virtual “general rehearsal” for the neurosurgical procedure to be performed. This ensures better communication, debate on the case, and idea-sharing, making the real surgical procedure optimized and safer (Figure 1).

### 2.5. 3D Printing

After the final STL file is obtained, the model can be materialized through the application of a 3D printer. Different types of printing material are available nowadays, depending on the model and the desired consistency, texture, color, and aspect of the object. The most-used materials include plastics, such as polylactic acid (PLA), acrylonitrile butadiene styrene (ABS), and polyethylene terephthalate glycol (PETG); resins like stereolithography (SLA) and digital light processing (DLP); or even ballistic gels and silicone, mainly used to replicate soft tissues.

Finally, the “surgery” can be practiced on the printed model, developing the manual skills required and considering the technical and anatomical nuances of the surgery and patient’s anatomy (Table 1).

## 3. Results

The described protocol has been developed in a progressive and original way in the Department of Neurosurgery of the Hospital General Universitario Alicante, Spain, with the collaboration and help of 3DNeurotrainer (www.3dneurotrainer.com) since October 2019, when we processed and produced the first real case. Since the beginning of the project, this technology has been applied in clinical practice to about 50 patients, and the 3D models have been used, both in VR systems and by 3D printing, on international training courses and for internal department training purposes.

To illustrate the benefits of this preoperative planning protocol, we will share the results obtained after applying it to five real cases operated at the Hospital General Universitario de Alicante.

### 3.1. Case 1: Sphenoid Wing Meningioma

**Figure 2:**
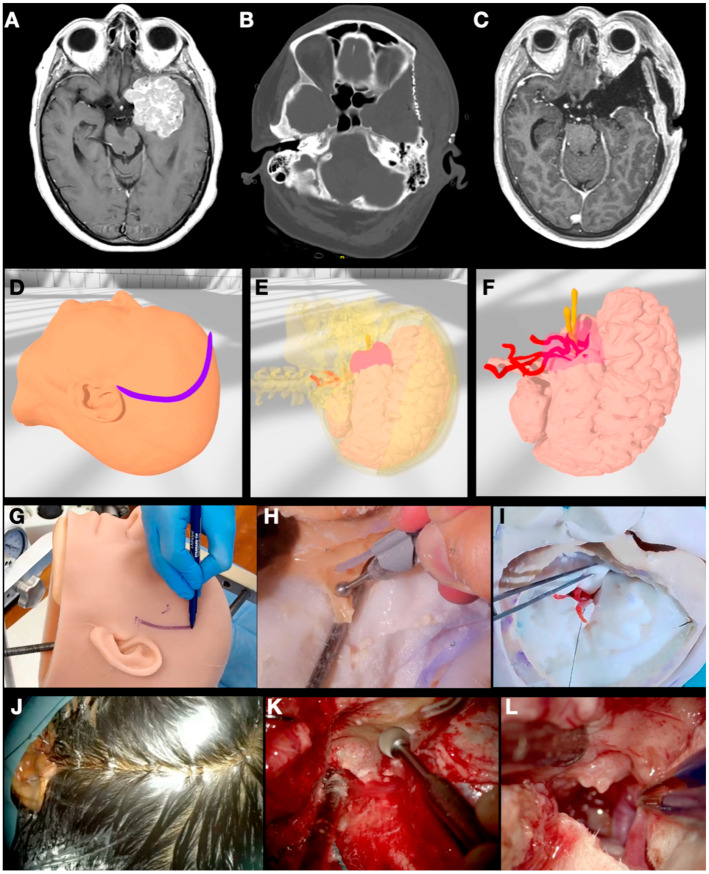
(**A**) Preoperative T1-GD-weighted MRI sequence from Case 1 showing a middle sphenoid wing meningioma on the left side. (**B**) Postoperative CT scan showing the intense sphenoid wing drilling necessary to create space and devascularize the tumor before dural opening. (**C**) Postoperative T1-GD-weighted MRI sequence. (**D**–**F**) Different images captured during collaborative VR planning, when the skin incision is designed, and the tumor is related to the skull, brain, and relevant neurovascular structures such as the internal carotid artery and the optic nerve. (**G**–**I**) Different steps of the surgical simulation over the 3D-printed model showing the skin incision marking (**G**), sphenoid wing drilling (**H**), and tumor capsule dissection of the middle cerebral artery (MCA) branches (**I**). (**J**) Skin incision on the real patient, followed by (**K**) sphenoid wing drilling, and (**L**) tumor dissection of the MCA bifurcation.

A 56-year-old woman consulted the emergency department after presenting an episode of tonic-clonic movements in the right hemibody with a subsequent loss of consciousness. A CT scan showed an intracranial mass related to the middle and anterior fossa, which was confirmed after carrying out an MRI. The imaging studies showed the suspected diagnosis of a left-side sphenoid wing meningioma with an important surrounding edema (Figure 2).

The previously described methodology was applied to the CT scan and MRI sequences. The objects “skin”, “skull”, “meningioma”, “brain”, “arteries”, and “veins” were segmented, modeled, and textured. Additional elements were created using the Blender^®^ software to simulate different sizes of craniotomy and sphenoid wing drilling. The idea behind these new objects was to evaluate the most appropriate craniotomy size for tumor and dural tail exposure as well as the need for intense skull base drilling with the aim of devascularizing the tumor and providing better access to its basal attachment. Different extensions and positions were practiced.

The segmented objects were then 3D printed and implemented into a head replica of the patient, composed of “skin”, “skull”, “meningioma”, “brain”, “arteries”, and “veins”. The dura was simulated by applying liquid silicone on the inner surface of the skull. The 3D printing accuracy was checked by uploading the patient’s preoperative images into StealthStation™ S8 navigation (Medtronic, North Ryde, NSW, Australia). After surface registration on the model, the target registration error was shown to be 0.7 mm after the first tracking.

Surgical simulation on the model was carried out by two neurosurgery residents and monitored by a consultant. A question mark frontotemporal incision was made. The pterion was then exposed and identified. Three burr holes were carried out, and the dura mater was dissected to perform the desired pterional craniotomy. The sphenoid wing was drilled out until the anterior clinoid process was reached cranially and the superior orbital fissure caudally. The dura was opened after checking the dural tail extension on the navigation system, and the tumor was progressively removed with an ultrasonic aspirator. The real surgery went smoothly without incidents, and the patient did well. The last six-month follow-up confirmed a complete resection of the mass.

### 3.2. Case 2: Pituitary Adenoma

**Figure 3:**
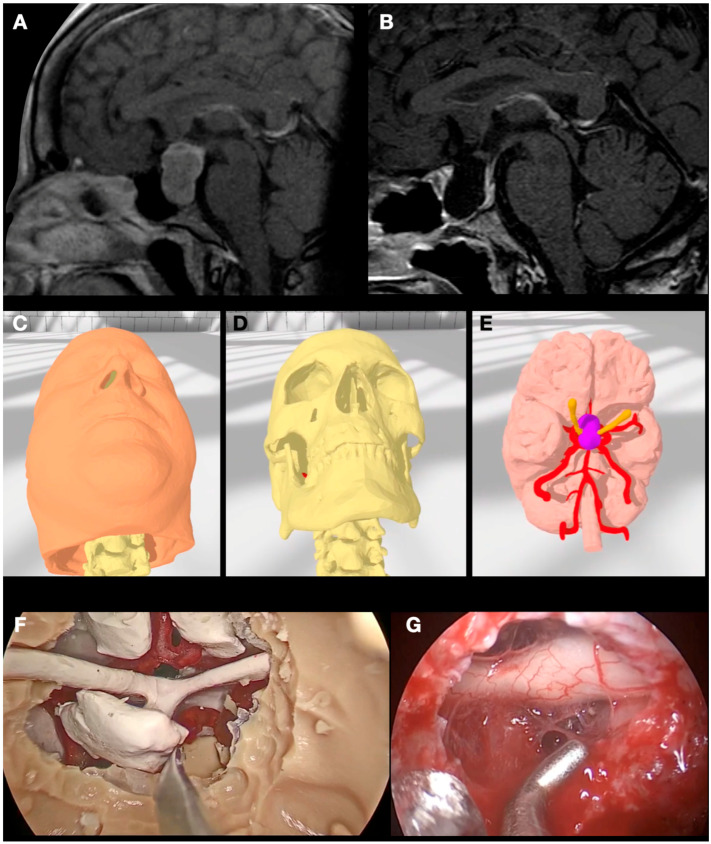
(**A**) Pre- and (**B**) post-operative sagittal slices on of the midline of Case 2 showing a pituitary adenoma. (**C**–**E**) Different steps of the collaborative VR surgical planning on the model itself showing the ideal head positioning as well as the relationship of the tumor to the optic nerve and internal carotid arteries. (**F**) View of the sellar and suprasellar spaces after drilling the sellar floor and removing the tumor on the model after simulating the surgery. (**G**) Same view on the real patient after tumor removal.

A 47-year-old man was referred to our outpatient clinic with the diagnosis of a pituitary macroadenoma. The patient started one week before with headaches and visual disturbances. On examination, the patient showed bitemporal hemianopsia. An MRI was performed diagnosing a pituitary macroadenoma with suprasellar and parasellar extension (Knosp 3A bilaterally) and chiasm compression (Figure 3).

A CT scan with contrast was also performed to complete the study. The segmentation protocol was applied to this CT scan to obtain “bone” and “carotid arteries”, while “tumor” and “optic nerves” were segmented from the MRI. After modeling and segmentation, additional sella turcica, the bone of the sphenoid rostrum, and sellar floor were segmented as isolated objects and created in the Blender^®^ software. The objective of this extra element was to determine the optimized lateral drilling to obtain the best tumor exposure without risk for carotid injury, maximizing the knowledge of the parasellar path of the latter.

The segmented objects were then 3D printed and implemented into a head replica of the patient, composed of the following: “skull”, “adenoma”, “brain”, “arteries”, and “optic nerves”. The nasal mucosa was simulated by applying pink liquid silicone on the inner surfaces of the nostrils and bilaterally over the nasal septum so the nasoseptal flap could be also practiced. 

Surgical simulation on the model was carried out firstly by one neurosurgery resident assisted by a consultant. Initially, a nasoseptal flap was planned and dissected, continued by a posterior septostomy. The sphenoid sinus was opened, confirming with neuronavigation the correct situation of the intrasphenoidal septa and both carotid arteries. When all the structures at risk were identified, the floor of the sella was drilled to reach the sellar dura, which was opened in an ‘X’ fashion. Finally, the tumor was aspirated including the exploration of both parasellar compartments, confirming adequate chiasm decompression. 

Finally, the real surgery was performed. It ran without incidences, with complete resection, and the patient’s visual impairment improved partially at the discharge time.

### 3.3. Case 3: Petrous Apex Meningioma 

**Figure 4:**
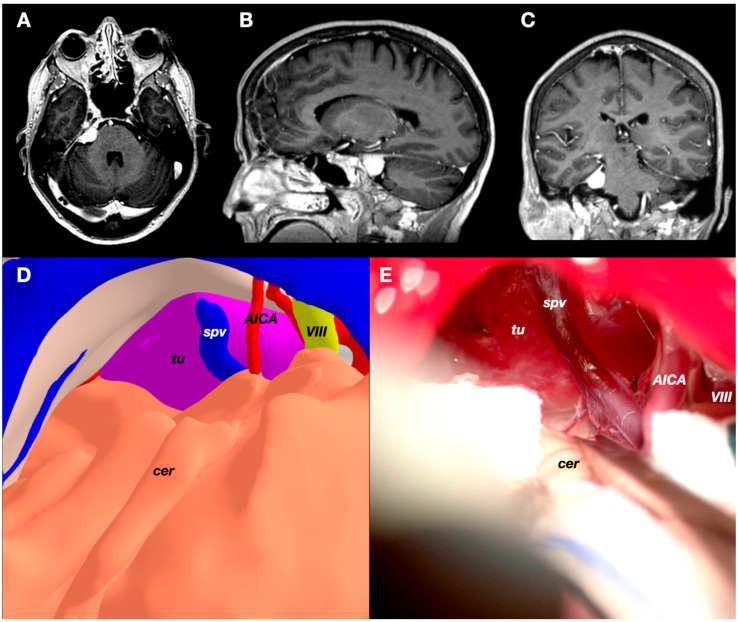
(**A**–**C**) Preoperative T1-GD-weighted MRI sequences of Case 3 showing a petrous apex meningioma on the right side. (**D**) VR 3D view where the cerebellum (cer), tumor (tu), superior petrosal vein (spv), VIII cranial nerve (VIII), and a distal branch of AICA (AICA) are shown. (**E**) Intraoperative view of the petrous apex meningioma showing the same neurovascular structures.

**Figure 5:**
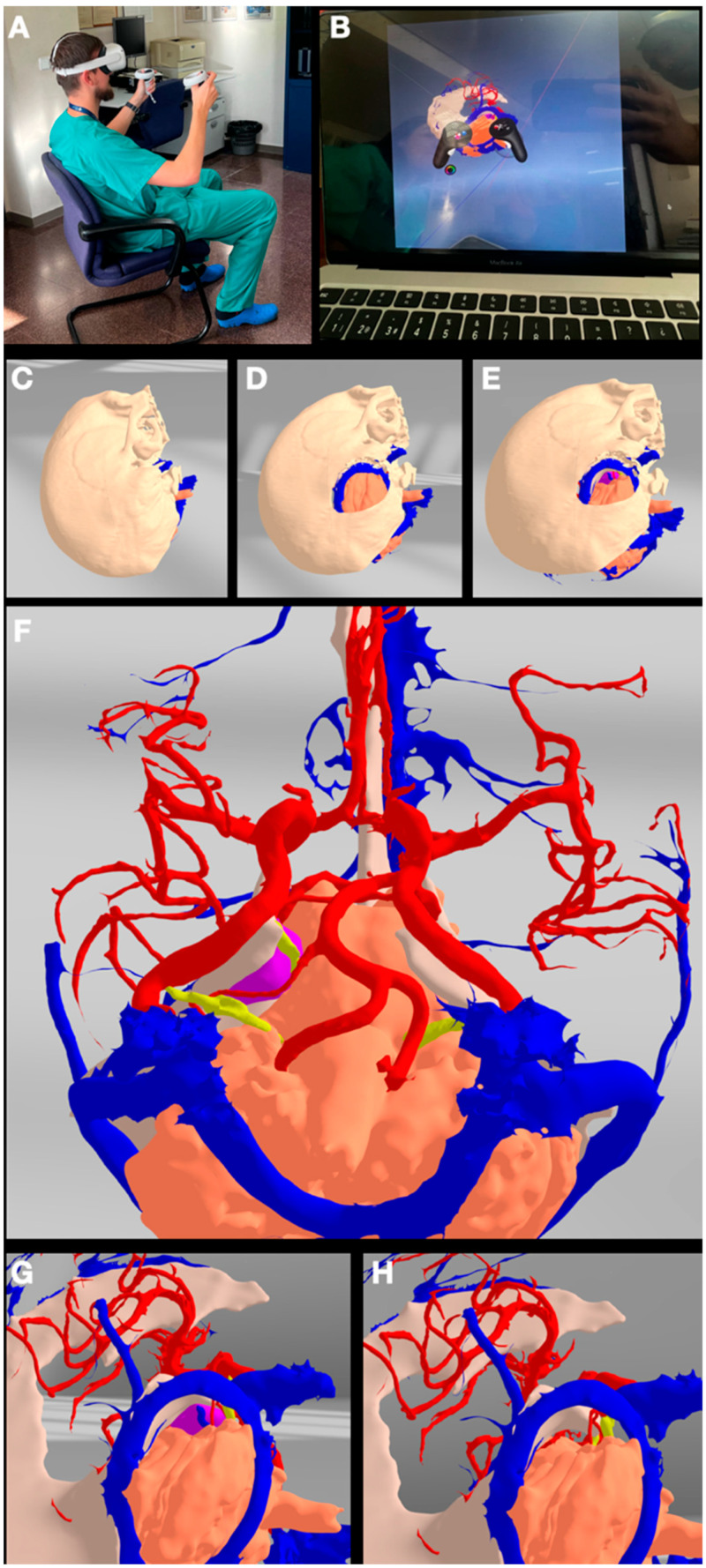
(**A**) Neurosurgical trainee practicing a retrosigmoid exposure from Case 3 on the VR system. (**B**) The consultant is monitoring the trainee’s virtual performance on a laptop, suggesting different maneuvers to be practiced live. (**C**–**E**) Different steps of the retrosigmoid craniotomy on the VR system. (**F**) Basal view of the patient’s anatomy where the tumor is colored in purple on the petrous apex area. Severe compression of the trigeminal nerve is noticed. (**G**,**H**) Surgical views of the retrosigmoid craniotomy after removing the bone and dura, with (**G**) and without the tumor (**H**).

A 52-years-old woman presented with atypical trigeminal neuralgia to the Neurosurgery clinic. The pain was resistant to carbamazepine and other different drugs. She had no other complaints, and the neurological examination was normal. A relatively small meningioma was identified in the MRI on the right petrous apex. The preoperative images were carefully studied, and the trigeminal nerve was identified partially in its cisternal segment. The fifth cranial nerve was compressed inferiorly and anteriorly by the tumor on its cisternal segment and at its entry point into Meckel’s cave. Due to the clinico-radiological presentation, surgery was offered (Figure 4 and Figure 5).

The objects “skin”, “skull”, “tentorium”, “petrosal veins”, “meningioma”, “cerebellum”, “arteries”, and “veins” were segmented, modeled, and textured using 3dslicer and Meshmixer. A standard retrosigmoid craniotomy was defined and separated from the skull through the Blender software. The transverse and sigmoid sinus were exposed after the craniotomy to increase access to the cerebellopontine angle (CPA) and the infratentorial space, as the tumor was attached to the petrotentorial junction at the level of the petrous apex. An additional object under the name “cerebellum” was created. In this second “cerebellum” element, the petrosal and tentorial surfaces were smoothed to simulate a retraction after opening the cisterns, releasing CSF, and introducing a cottonoid into this area. After introducing all these data into the VR system, the head was placed in a lateral position, and the retrosigmoid craniotomy was removed. This allowed the visualization of the tumor attached to the dura of the tentorium and the petrous apex. Additionally, VR allowed us to understand the relationship between the tumor and the surrounding neurovascular structures. Therefore, a transverse pontine vein draining into the superior petrosal vein complex was identified running just posterior to the tumor between our surgical trajectory and the tumor itself through the CPA route. Moreover, the AICA distal branch was shown to be just under the tumor.

These findings changed somehow our initial plan, so that our first maneuver consisted of avoiding the route through the CPA, first accessing the infratentorial supracerebellar compartment to devascularize and debulk the tumor from its tentorial attachment, with the aim of respecting the transverse pontine vein. Although this model was not printed, the VR practice and views allowed us to visualize and imagine the surgical field days before the procedure itself, modifying our initial strategy. The tumor was removed completely, and the patient’s pain disappeared. She only complained of a mild hypoesthesia in the V2 territory of the trigeminal nerve.

### 3.4. Case 4: Foramen Magnum Meningioma 

**Figure 6:**
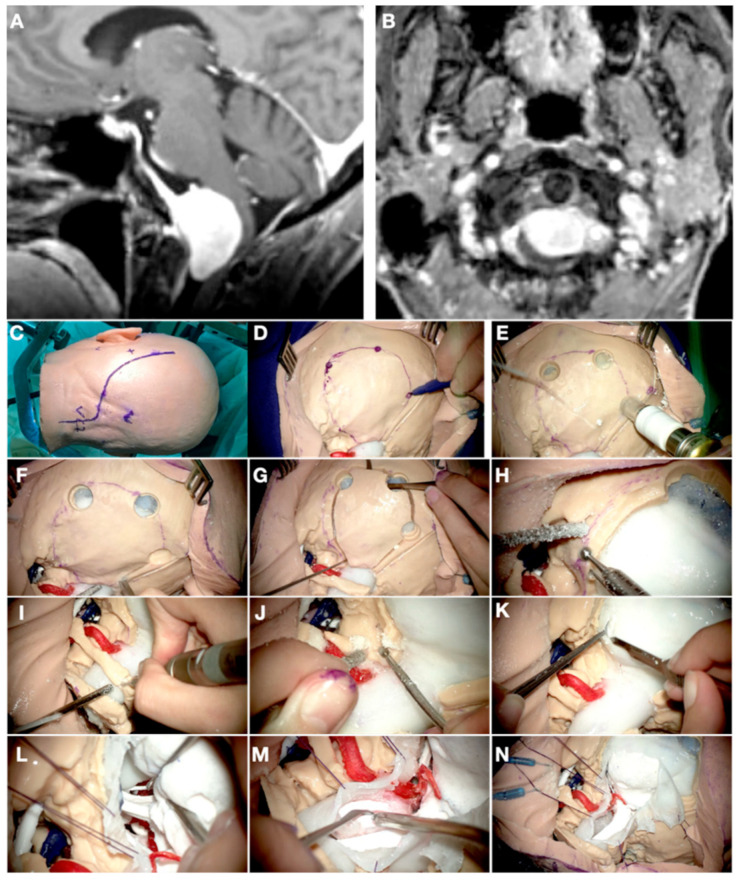
Different steps of the surgical simulation on Case 4 (left-side foramen magnum meningioma). (**A**,**B**) Preoperative sagittal and axial T1-GD-weighted MRI images of the patient. (**C**) Head clamping and patient positioning. (**D**) Craniotomy design. (**E**) Burr holes on the transverse sinus. (**F**) Far-lateral craniotomy. (**G**) Dural dissection and bone flap elevation. (**H**) Posterior mastoid drilling. (**I**) C1 posterior arch removal. (**J**) Condyle drilling. (**K**) Dural opening posterior to the sigmoid sinus. (**L**) Cerebellopontine angle exposure showing the VII–VIII and lower cranial nerves as well as the PICA. (**M**) Tumor debulking. (**N**) Final view before dural closure.

A 73-years-old woman was referred to our clinic with the suspected diagnosis of a medium-size foramen magnum meningioma. She complained of a slow progressive loss of lower extremity strength with an uncomfortable feeling of unsteadiness when walking. A left-sided foramen magnum tumor was shown on the CT scan and preoperative MRI, compressing the medulla oblongata posteriorly and to the contralateral side (Figure 6).

The objects “skin”, “skull”, “tentorium”, “meningioma”, “cerebellum”, “brainstem”, “cranial nerves”, “arteries”, “venous sinuses”, and “veins” were segmented, modeled, and textured using 3dslicer and Meshmixer. A lateral suboccipital craniotomy exposing the sigmoid sinus laterally was defined, and the left C1 hemilamina was also included in it. Both new elements were separated from the skull through the Blender software and named “far-lateral craniotomy”. Different extensions of occipital condyle drilling were tested. Obviously, each millimeter of condyle resection offered a bit more ventral space to access the tumor attachment. However, moving the lateral extension of the craniotomy to the condyle without any resection on it was shown to be enough to reach the most lateral attachment of the tumor.

The different components of the model were 3D printed and implemented to simulate the surgical field. The model was finally placed in a right lateral position with the head slightly rotated to the left side. This allowed a theoretical relaxation of the cerebellum after opening the dura, providing extra space to access the tumor attachment and the surrounding neurovascular structures. A sigmoid incision was made, and a large lateral suboccipital craniotomy was performed. The most lateral part of the occipital bone was drilled away as well as the posterior mastoid to skeletonize the posterior aspect of the sigmoid sinus towards the condyle. The ipsilateral C1 arch was removed to gain basal space. The dura was then opened also in a sigmoid shape, following the lateral limit of the craniotomy. The spinal accessory nerve and vertebral artery were visualized just posterior and basal to the tumor, forcing the first maneuver to be made superiorly, into the tumor attachment to the foramen magnum dura.

The VR and surgical simulation gave us some good tips about the positioning of the patient as well as the kind of craniotomy and skull base drilling to be performed before opening the dura in this case. The surgical procedure went uneventfully, and the patient was discharged after checking for a complete resection in the postoperative scan and MRI. Three months after surgery, the patient reported a resolution of her motor symptoms.

### 3.5. Case 5: Falcine Meningioma 

**Figure 7:**
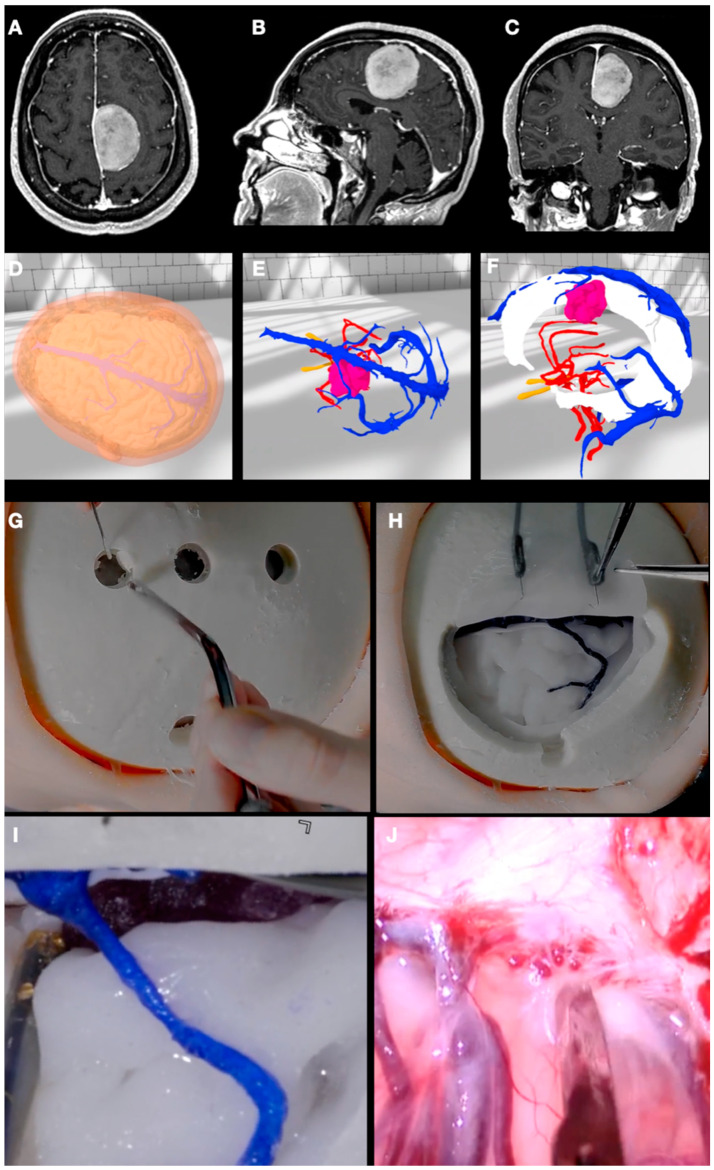
(**A**–**C**) Preoperative T1-GD-weighted MRI sequences of Case 5 showing a left-side parafalcine meningioma. (**D**,**E**) VR views after placing the model in a left lateral position to simulate the surgical field. The close relationship with the venous midline structures is shown. (**F**) Left lateral view of the patient’s anatomical structures after having obscured the skin, skull, and brain layers. The relationship of the tumor with the superior sagittal sinus and a frontal bridging vein is clearly understood. (**G**) Surgical simulation on the model after having performed three midline burr holes. (**H**) Surgical simulation on the model after dural opening. The frontal bridging vein is clearly identified. (**I**) A magnified view shows the location of the tumor in the interhemispheric fissure related to the frontal vein. (**J**) The intraoperative view confirms the surgical planning and simulations.

A 55-years-old woman was referred to our clinic to evaluate a parafalcine meningioma discovered after the study of a single partial verbal seizure. She also complained of subjective difficulty in reading comprehension and writing. The rest of the neurological examination was irrelevant. An MRI was performed, objectifying a parafalcine meningioma with compression of the medial frontal lobule (Figure 7).

The objects “skin”, “skull”, “cortical veins”, “superior sagittal sinus”, “meningioma”, “brain”, and “arteries” were segmented, modeled, and textured using 3dslicer and Meshmixer. A frontoparietal craniotomy including the coronal suture centered above the tumor convexity extension was defined and separated from the skull through the Blender software. VR analysis and planning were performed in the “virtual room” by two neurosurgical trainees and a consultant, who discussed the positioning and extent of the craniotomy and the relationship of the tumor with two frontal bridging veins draining into the superior sagittal sinus. Finally, a neutral supine position with mild extension of the head was decided. A wide-enough craniotomy that included the two bridging veins and an interhemispheric dissection in-between the veins were chosen as the best surgical route.

The elements “skin”, “skull”, “cortical veins”, “superior sagittal sinus”, “meningioma”, “brain”, and “arteries” were 3D printed and assembled for surgical simulation and practice. The surgery was practiced on the model as planned, and, later, the patient was operated on without any unexpected events. Two years after surgery, the patient is doing well, not taking antiepileptic drugs and being free of seizures after complete tumor resection.

## 4. Discussion

The success of any given surgical procedure in neuro-oncology depends on numerous factors that can influence clinical outcomes for a patient. The most relevant intrinsic preoperative factors include tumor type and grade, size and location, patient’s overall health, neurological status at diagnosis, and presence of swelling and peritumoral edema as well as vascular and blood supply. Other intra- and postoperative variables, such as the extent of the resection, the use of advanced technology like neuromonitoring, neuronavigation, visualization, and intraoperative imaging techniques as well as postoperative care and rehabilitation complete this complex landscape [25,26]. Communication with the patient and adequate management of potential complications is also essential for the success of a surgery.

To summarize, all these variables can be divided into factors intrinsic to a case, in which no action involves a direct impact on the result, and those on which the physician’s intervention can change the clinical course of the patient, thus making the difference between success and failure. Regarding the latter, irrespective of their nature, they all depend in some way on the surgeon’s experience. Surgeons’ expertise plays a critical role in various aspects of a surgery, including the following: the choice of the most appropriate approach, the surgical technique and manual skills, decision-making during surgery (ability to take fast decisions and actions to deal with unexpected findings), minimizing the damage to surrounding tissues to preserve and/or improve the neurological function, a better use of advanced technology, and, probably the most important one which could include all the already-mentioned factors, the ability to mentally create a step-by-step plan, including the ability to anticipate any event that deviates from the expected course and provide a quick solution to allow one to return to the plan, and, if it is not possible, to continue with an alternative plan. All these aspects should be listed at different extents in the so-called surgical planning [27,28].

### 4.1. Surgical Planning

Preoperative surgical planning is a mental exercise that consists of a step-by-step description of all the events that a surgeon aims to perform during a procedure, having previously studied the preoperative images as well as considering the anatomy of the area in which the work is going to be carried out. A strong anatomical knowledge, analysis of biplanar images, and their mental transformation to 3D images is fundamental for the surgeon to be able to reach an idea of the surgical field to be encountered even before making the incision.

The advantages provided by our protocol to enhance the surgical planning process are many and, specifically, based on better anatomical 3D comprehension and the possibility of training on patient-specific lesions. We can never forget cadaver dissection for 3D imaginary acquisition, which remains essential in studying normal anatomy and understanding tissues’ behaviors as well as the relationship between different anatomical structures; however, the brains used are usually normal brains without pathology. The described method is complementary to the anatomical studies on cadavers and provides different tools for neurosurgeons. Indeed, this model is based on the real anatomy of a patients since it is created from the actual images of each subject. Thus, it provides a more realistic patient-specific simulation, helping surgeons understand the displacement of the structures caused by tumor mass effect as well as design specific surgical approaches and maneuvers to be performed.

### 4.2. Virtual Reality

VR technology is nowadays being implemented not only in medicine but also in many other different fields. This new armamentarium allows one to create ideal environments for training and education and work cooperatively on various projects. This growing trend has entered medicine and, especially, the surgical fields and seems to be here to stay and further develop. Some groups have published papers on its potential uses in neurosurgery [3,4,5,6,7,12,13,14,15,16,29,30,31,32,33,34,35,36,37,38,39,40,41,42,43,44,45,46,47,48,49,50,51,52,53,54,55,56], but none of them has reported a step-by-step protocol including all the different parts of the segmentation and modeling processes for each 3D object.

Our protocol shows this kind of step-by-step procedure to create 3D models of a patient’s neuroanatomical structures. These objects can be “sculpted” by managing DICOM images in 3D Slicer. This software permits the segmentation of all the different tissues (brain, tumor, arteries, veins and sinuses, cranial nerves, skull, and skin, among others) based on preoperative brain MRIs and CT scans by exporting them into 3D objects to work with. Later, by using the Meshmixer and Blender software, these objects can be refined and texturized, giving them a more realistic appearance. By loading all the reconstructed structures in any VR system, the representation of the patient’s anatomy and pathology can be studied in full detail. Placing the head of the patient in the surgical position, the surgical approach and craniotomy as well as the anatomical corridor to the lesion with its structural nuances can be visualized and studied in VR any time before surgery, serving as a “general rehearsal” for the surgeons and the trainees.

The visualization of surgical cases in VR can provide a whole new variety of benefits in different aspects of the neurosurgical field. Some of the advantages found in VR are the ability to zoom in and out and see a patient’s specific anatomy from any angle and the possibility of disassembling the model without destroying it, so that it can be used as many times as needed, among others. The multiuser modality of this type of visualization can enhance surgical planning as it makes communication between surgeons and surgical trainees easier [7]. This can be performed independently of the geographical location of the surgeons and/or trainees, opening lots of possibilities in terms of global neurosurgery, both in surgical planning (by discussing the case) but also from an educational perspective [42].

VR simulators have been shown to improve the learning curve in some neurosurgical procedures, like, for example, neuroendoscopy [12], assessing interpersonal skills and technical skills’ evaluation [32], lateral ventricle puncture [38], etcetera. Other benefits of using VR in neurosurgery have been described in the growing literature in recent years. Benefits in training (manual dexterity), planning (patient-specific environment), education (neuroanatomy learning, spatial relationships between structures), surgical navigation (“see-through” anatomy visualization), safety (training in a safe environment), therapeutics (rehabilitation in neurological patient’s conditions), and communication (better relationship between the surgeon and the patient) have been cited [49].

VR models can also be implemented in neurovascular cases, allowing for a better visuospatial localization of an aneurysm’s projection, shape, and relationships to the surrounding structures [7]. This permits VR planification of complex surgeries and the ability to elucidate operative prognostication and anticipate decision making during surgery [6]. Zaed et al. have even proven a reduction in the time needed to perform aneurysm clipping when a preoperative study was carried out through VR technology [37].

In spine surgery, numerous benefits of using VR and AR have been described, among which are improvements in surgical planning, training, and patients’ rehab. It is important to note that AR has more uses during surgery in comparison with VR, which usually serves as a preoperative tool. The implementation of AR during surgery has been shown to reduce X-ray use and allow more accuracy in screw placement than freehand techniques and better rod bending and percutaneous vertebroplasty guidance, among others [34]. 

VR has also been used for educational purposes in morphometric investigations. Cadaveric specimens have been scanned and the DICOM images obtained reconstructed in a VR model. This can open the door to studying cadaveric dissections in other places, distinct from the anatomy lab [3].

Another interesting potential use of VR is to increase communication between the patient and the surgeon. Preoperative interviews and discussions with patients may be carried out through a VR system with the aim of achieving a better understanding of their disease and making informed decisions in the interest of their health [3].

However, some limitations in terms of VR uses should be highlighted, like cost implications, the limited graphics processing capacity of some devices, and cybersickness, which usually consists of nausea and vomiting, vertigo, or headaches during or after a VR experience [39].

### 4.3. 3D Printing

Due to the advancing technology and its increasing availability, the use of 3D printing technology has emerged in various fields of neurosurgery [8,9,10,11,57,58,59,60,61,62,63,64,65,66,67,68,69,70,71,72,73,74,75,76,77,78,79,80,81,82,83]. As 3D technology goes further, the costs of printers and materials lower while printed materials become more realistic, making the application of 3D printing in the medical field more appealing [11]. Three-dimensional printing in a multi-material manner has been shown to be a useful preoperative tool for preoperative planning, combining both cost-effectiveness and flexibility to be utilized for patients’ benefit and medical residents’ education [58]. 

Studying human neuroanatomy is not an easy task. Classically, this has been achieved by studying from 2D textbooks and from cadaveric dissections, which is still considered the gold standard [49]. However, not all surgical trainees have the possibility to access anatomical specimens due to their high costs, their maintenance as well as a shortage of cadavers [8]. The method described earlier in this paper can somehow solve this problem, providing the possibility to use patient-specific anatomy for both teaching and planning purposes [75]. Simulation of surgical procedures and training may improve clinical results and surgeons’ competence [58]. Another advantage of this is the possibility to gain surgical skills by performing surgical approaches for tailored neurosurgical diseases that can be presented in a regular manner, through 2D images from CT scans and MRIs.

The use of 3D-printed models in neurosurgery has been studied in several fields. Langdon et al. explored the use of a 3D-printed model of a skull base tumor in a pediatric patient, finding benefits in terms of surgical planning and training [58].

The application of 3D printing in the field of vascular neurosurgery has been described by several authors. A 3D-printed vasculature was applied to education, training, and presurgical planning, including patients’ anatomic variants, which allowed for better decisions regarding surgical candidates, selecting the optimal approach, and decreasing the operative time and the rate of complications [71]. Wang et al. described the use of 3D-printed vascular anatomy and pathology from 3D-DSA for preoperative studies and analyses. Residents and trainees answered a questionnaire that showed that these models helped them understand the shape, location, and direction of the aneurysm, the size and type of clip that needed to be used, and the parent artery of the aneurysm and improve their medical–patient communication [61]. In the study of Li et al., the application of 3D printing models in the treatment of aneurysms through an eyebrow keyhole approach was explored. In one group, preoperative planning was performed just with 2D images and, in the other, with a curved multiplanar reconstruction and a 3D-printed vascular model of the patient’s anatomy and pathology. The results showed that 3D curved multiplanar reconstruction images combined with 3D printing technology can achieve better surgical and imaging results in terms of intraoperative time, area of surgical exposure, Glasgow Outcome Score (GOS) scale, and postoperative complications [68].

Huang et al. studied the application of 3D printing models of macroadenomas in endoscopic endonasal surgery, finding benefits in having less operation time and blood loss as well as lower postoperative complications rates. The authors concluded that 3D-printed models are not only useful for presurgical practice but can also provide valuable assistance in making surgical decisions [65]. Gillett et al. explored the different materials of 3D printing methods in pituitary tumors, being the vat photopolymerization preferred by neurosurgeons due to its consistency and similarity to the real tissue [73].

Peng et al. explored the combined use of a 3D-printed model and mixed-reality (MR) glasses to train in the ventricular puncture technique as well as to complete endoscopic trajectory training for basal ganglia intracerebral hematomas (ICHs). The authors compared results between two groups, one without MR and the other with MR, showing that the second group achieved better results in terms of positioning, depth of puncture, accuracy, and self-confidence, among other criteria [10].

In the article by Dho et al., the authors described a workflow from DICOM images acquisition to a 3D-printed brain tumor model and its potential benefits, close to our flowchart for creating the printed 3D examples. There was a significant benefit in terms of surgical position and craniotomy design, and, due to the preoperative study and dissection of the implemented gyri and sulci, participants could determine the most effective cortical window to the patient’s tumor, avoiding damaging eloquent areas. The benefits were greater in the group of less experienced surgeons [9].

There have been numerous uses of 3D printing in spine surgery, from valuable benefits obtained from surgical planning to customized implants providing better stability [57]. Another interesting use of 3D printing was described in spinal deformity surgery by printing drill templates that increased the accuracy of pedicle screw placement [77]. 

An important factor that is worth mentioning is that several articles describe a benefit from using 3D-printed models in terms of doctor–patient relationships as well as communication with the latter’s family. The patient’s disease can be shown on the 3D-printed model, and intricate explanations given to both the family and the patient themselves, usually without medical education, are better understood, and the decisions made by the patient are more informed [58].

Some of the limitations of 3D printing are the duration of the printing process, registration and segmentation inaccuracies, as well as drift possibility, making a 3D model less accurate [10]. Indeed, even though all our cases were segmented and implemented into the VR system to be analyzed and facilitate surgical planning, only a few of them were fully 3D printed. Due to the necessity to invest 2–4 days for printing, we decided to 3D print only those cases that were representative of a common disease. Therefore, we created 3D-printed models for classic lesions such as sphenoid wing meningiomas, convexity tumors, pituitary adenomas, vestibular schwannomas, and so on.

### 4.4. Limitations

The main limitation of our protocol is its high cost in the case of printing, reaching over one thousand EUR depending on the difficulty and details of the model to print. If printing is not necessary, our protocol is quite affordable because all the software utilized here are open-source free code, with VR glasses being the only expense, which can be affordable, starting from about 300 EUR.

The other limitation of our protocol is the need for a learning curve and difficulties due to the images’ quality. The first cases, especially if a model was going to be printed as more postprocessing is needed to smooth it, took us more than 12 h to be ready. However, in the most recent cases, we could obtain a good model for VR training in about three to four hours. That is why we recommend starting with simple cases (such as convexity meningiomas or simple cortical gliomas) to familiarize oneself with all the needed software and, afterwards, increase the difficulty by moving onto studies of skull base tumors, more complex gliomas, and even neurovascular diseases such as aneurysms and arteriovenous malformations.

Finally, the main limitation of this study, as this is not within the scope of our present descriptive work, is that no objective measures were included regarding the models’ accuracy (mm distance between structures in a model and in the real surgery setting) or time-saving procedures. Subjectively, we found this protocol worthwhile due to its accuracy and ability to change predetermined plans, but more studies are needed to obtain a full objective picture of the utility of this protocol.

## 5. Conclusions

Surgical procedures in neuro-oncology require the surgeon to carry out a detailed and individualized study of the preoperative images of each case. Historically, this analysis has been referred to as surgical planning and has been performed by studying a stack of two-dimensional images. Continuous development and technological advances have allowed this stack of images to become a combination of different three-dimensional structures, making their analysis more intuitive.

Our original protocol allows for the implementation of all these three-dimensional objects in a virtual reality space where they can be modified (changes in size, position, transparency, perspective, etc.) so that surgeons can study each case individually and from a much more immersive perspective to develop much safer surgical plans. In addition, these objects can be printed, forming an exact replica of the anatomical structures of the patient to be operated on so that the surgical team can perform an advanced simulation before the actual surgery.

Further studies will be needed to assess the usefulness and accuracy of these technologies.

## Figures and Tables

**Table 1:** Step-by-step guide to perform 3D VR surgery rehearsals and obtain printed models.

Step 1	Patient image acquisition (CT, CTA, MRI, etc.)	
Step 2	Download the DICOM images	
Step 3	Load the DICOM images into 3D Slicer	
	Open the “Segment Editor” module: the “Threshold”, “Draw”, and “Grow from seeds” tools, among others, are used to segment the neuroanatomical structures	
	Export files to an .STL format	
Step 4 (*)	Load the .STL file into MeshMixer	
	Use the “Inspector” and “Sculpt” tools, among others, to refine the reconstructed radio-anatomical structure	
	Export the refined object into an .OBJ file	
	**VR**	**3D printing**
Step 5 (*)	Open the .OBJ file with Blender	Select the desired material: polylactic acid, acrylonitrile butadiene styrene, polyethylene terephthalate glycol; resins like stereolithography; digital light processing; and ballistic gels, among others
	In the “Material” menu, select the desired color and texture	
	Export the file to an .FBX format	
Step 6	Import the reconstructed structures to Gravity Sketch on a Meta Oculus Quest 2 VR headset	Print the reconstructed structures
	Assemble all the desired structures into the VR model	Assemble all the printed structures into the model
Step 7	The surgical case can be studied, analyzed, and detailed in VR	The surgical case can be performed, trained, and studied hands-on on the printed model

* Note that steps 4 and 5 are repeated for all the previously segmented and exported .STL files.

## Data Availability

The raw data supporting the conclusions of this article will be made available by the authors on request.
